# New Window into Breast Cancer Risk: Assessing Lifetime Exposures to POPs

**DOI:** 10.1289/ehp.116-a307a

**Published:** 2008-07

**Authors:** M. Nathaniel Mead

Persistent organic pollutants (POPs) such as polychlorinated biphenyls (PCBs) are ubiquitous chemical compounds that persist in the environment and bioaccumulate through the food web. Although experiments have shown that POPs stimulate the proliferation of human cancer cell lines, epidemiologic studies of POP-associated cancer risk have yielded inconsistent results, possibly because of the lack of tools for estimating lifetime exposures to these chemicals. Now, however, researchers have developed a new physiologically based pharmacokinetic (PBPK) modeling approach that can potentially be used in epidemiologic studies to simulate lifetime toxicokinetics of POPs in women **[*EHP* 116:886–892; Verner et al.]**.

Previous biological assessments have been limited to measuring POP levels in blood or tissue samples collected around the time of breast cancer diagnosis. However, such assessments may not reflect the body burden during earlier, potentially critical exposure points in a woman’s life such as the fetal, postnatal, and adolescent periods.

In contrast, the new model integrates the relevant processes of absorption, distribution, metabolism, and elimination to estimate lifetime blood and tissue exposure and levels during any hypothesized time window of susceptibility in breast cancer development. The model also predicts how various types of relevant lifetime physiologic changes—such as body weight variation, pregnancy, excretion of POPs through lactation, and aging—will influence the kinetics of a compound in a woman throughout her life. The model enables the estimation of interindividual differences in POP exposures through the use of physiologic information obtained from questionnaires in epidemiologic studies.

The researchers found that lactation and weight change histories had the greatest impact on the toxicokinetic profile throughout life. According to the model, the longer and later in life lactation occurred, the lower the woman’s blood POP concentration at age 55 (a surrogate time representing the typical age at breast cancer diagnosis). Similarly, variations in body weight throughout life had a greater impact than average body weight on blood POP concentrations, possibly because weight loss is accompanied by unloading of POPs into the blood via lost adipose tissue. This means that quantitative information on both lactation and body weight histories is critical when evaluating past POP exposures.

If, as some researchers hypothesize, breast cancer is related to POP exposures at specific time windows of susceptibility during a woman’s lifetime, lactation and body weight histories must be considered in studies of POP exposures and breast cancer risk. Depending on when such physiologic events occur, women having similar POP concentrations at the age of diagnosis may have had completely different internal levels at a time that may be critical to the formation of breast cancer. The proposed PBPK modeling approach therefore could be used in environmental epidemiology research to circumvent limitations inherent in relying on late-life sampling for past exposure assessments.

